# Editorial: Preventing multiple sclerosis

**DOI:** 10.3389/fneur.2022.982411

**Published:** 2022-07-20

**Authors:** Ruth Dobson, Radu Tanasescu, Bruno Gran

**Affiliations:** ^1^Preventive Neurology Unit, Wolfson Institute of Population Health, Queen Mary University London (QMUL), London, United Kingdom; ^2^Department of Neurology, Royal London Hospital, London, United Kingdom; ^3^Department of Neurology, Nottingham University Hospitals NHS Trust, Nottingham, United Kingdom; ^4^Mental Health and Clinical Neurosciences Academic Unit, School of Medicine, University of Nottingham, Nottingham, United Kingdom

**Keywords:** multiple sclerosis, prevention, risk factors, causation, early diagnosis

Multiple sclerosis (MS) prevention has been identified as a key aim across MS research. Achieving this aim is complex—MS is relatively rare, there is a long lag between many identified risk factors and clinical MS development, and many people exposed to these risk factors never develop MS. It is clear that MS has a complex pathogenic pathway with contributions from, and interactions between, genes and environment.

In this Research Topic, we present a group of papers which seek to explore opportunities and challenges around MS prevention, and how some of these may be overcome. Tremlett et al. discuss whether further exploration of the MS prodrome, which can be detected years prior to clinical MS diagnosis, could enhance prevention efforts. By identifying those at the earliest stage of disease, prior to the development of neurological disability, they argue that there needs to be a re-evaluation of the risk factor literature, such that reverse causation during the prodromal period can be considered. A greater understanding of both true risk factors, and factors acting on disease progression during the prodromal period has the potential to inform prevention and early disease modification prior to neurological symptom onset. Pediatric MS has a complex aetiological pathway, with similar environmental risk factors identified to adult onset MS. Hardy et al. argue that investigating environmental determinants in this population overcomes at least some of the challenges associated with adult onset MS, given the closer temporal association between risk factor exposure and disease onset.

One of the environmental factors consistently associated with MS development in epidemiological studies is vitamin D deficiency. However, reverse causation and lack of direct mechanistic evidence remains a concern. Haindl and Hochmeister review the evidence from animal models, particularly mouse models, which provide potential mechanistic insights around inflammatory disease, but add little to our knowledge around the impact of vitamin D on progression. They highlight limitations of such studies, including around dosage, the potential anti-inflammatory role of UV light, and differing biology between EAE and MS. Gombash et al. review whether vitamin D acts *via* immunoregulatory or direct neuroprotective mechanisms. Immunological mechanisms have been demonstrated in both animal models and human studies; vitamin D appears to have actions on both the innate and adaptive immune systems. There is also substantial evidence that vitamin D acts as a neurosteroid, with the vitamin D reception expressed throughout the developing and mature brain.

Infection with Epstein Barr virus has been identified as a potential obligate step in MS development. Hassani et al. demonstrate, *via* a rabbit model, that primary peripheral EBV infection can lead to the virus traversing the CNS within the cells it infects. Within the CNS, B lymphocytes develop inflammatory cellular aggregates, the first direct *in vivo* evidence for the role of peripheral EBV infection in CNS pathology. Targeting EBV in order to prevent MS requires an understanding of the most appropriate means to tackle and potentially prevent infection. Kearns reviews the mathematical and biological underpinnings of gene-environment interactions with a focus on EBV, and discusses the implications for EBV-focussed prevention interventions. Vaccination, anti-virals, immunotherapies and cell-based therapies are all discussed as potential strategies. Maple et al. take this a step further, with an in depth discussion around EBV vaccination. The authors highlight the two current approaches around vaccination—either a prophylactic vaccine to prevent infection or disease, vs. a therapeutic vaccine to treat people with EBV-associated complications such as cancers. They argue that an EBV vaccine to prevent IM is the most likely strategy to be tested and adopted, but that vaccine hesitancy and high seroconversion in early childhood are important considerations in any vaccine rollout.

Smoking prevention strategies are well-established in the wider public health literature. Whilst many of these strategies focus on non-MS health consequences of smoking, such as cancer and vascular disease, the contribution of smoking to MS development should not be overlooked. Manouchehrinia et al. use a large population-based cohort to determine the population attributable risk associated with smoking, taking into account HLA type. They demonstrate that the overall attributable fraction of MS associated with smoking is 13.1%, with a higher point estimate in males (19.1%) than females (10.6%), and that approximately half of the attributable fraction due to smoking is independent of HLA-associated risk. This highlights the importance of smoking prevention and cessation efforts in terms of MS prevention.

Understanding transcriptomics may help to understand how the risk factors highlighted in this Research Topic influence disease development. Elkjaer et al. review the existing literature on transcriptomic studies within the CNS. They find support for MS as a whole brain disease, with inflammation, iron disturbances, cellular stress, and hypoxia. They show heterogeneity within MS at molecular level, contrasting with the relative clinical homogeneity. This provides insight into some of the complexities with MS prevention efforts—targeting a highly heterogenous disease is likely to require multimodal interventions.

The collection of articles within this Research Topic demonstrates that for prevention efforts to have clinically meaningful impact, identifying those at highest risk is key. One potential strategy is secondary prevention at the earliest disease stage. Amato et al. discuss predictors of evolution in those with radiologically isolated syndrome, the earliest clearly defined stage of MS. Disease modification studies are currently being performed at these earliest stages, however these are using licensed MS therapies rather than targeting modifiable risk factors.

In order to move the prevention therapeutic window earlier, Hone et al. highlight some of the challenges associated with MS risk scores, including that performance metrics fall well short of those required for a diagnostic or predictive test. Incorporating cross-ancestry portability and environmental factors are key considerations for clinical use. However, whilst we are unlikely to be able to predict MS on an individual basis in the near future, risk scores may be useful to identify high-risk populations for preventive trials, such as EBV vaccination.

This Research Topic therefore demonstrates that there is sufficient evidence to support action to trial interventions to prevent MS, with clearly defined interventions in selected high risk populations being key to success. Without the courage to set up studies to understand the impact of interventions, the need for prevention will remain unaddressed—this cannot continue given the importance of this Research Topic.

## Author contributions

RD: initial drafting of manuscript and approval of final manuscript. RT and BG: critical review, input into manuscript, and approval of final manuscript. All authors contributed to the article and approved the submitted version.

## Funding

RD works on the Preventive Neurology Unit, which was supported by Barts Charity. RT received support from the UK MRC (CARP MR/T024402/1).

## Conflict of interest

The authors declare that the research was conducted in the absence of any commercial or financial relationships that could be construed as a potential conflict of interest.

## Publisher's note

All claims expressed in this article are solely those of the authors and do not necessarily represent those of their affiliated organizations, or those of the publisher, the editors and the reviewers. Any product that may be evaluated in this article, or claim that may be made by its manufacturer, is not guaranteed or endorsed by the publisher.

